# Social interactions promote adaptive resource defense in ants

**DOI:** 10.1371/journal.pone.0183872

**Published:** 2017-09-14

**Authors:** Christoph Johannes Kleineidam, Eva Linda Heeb, Stefanie Neupert

**Affiliations:** Behavioral Neurobiology, Department of Biology, University of Konstanz, Konstanz, Germany; University of California San Diego, UNITED STATES

## Abstract

Social insects vigorously defend their nests against con- and heterospecific competitors. Collective defense is also seen at highly profitable food sources. Aggressive responses are elicited or promoted by several means of communication, e.g. alarm pheromones and other chemical markings. In this study, we demonstrate that the social environment and interactions among colony members (nestmates) modulates the propensity to engage in aggressive behavior and therefore plays an important role in allocating workers to a defense task. We kept *Formica rufa* workers in groups or isolated for different time spans and then tested their aggressiveness in one-on-one encounters with other ants. In groups of more than 20 workers that are freely interacting, individuals are aggressive in one-on-one encounters with non-nestmates, whereas aggressiveness of isolated workers decreases with increasing isolation time. We conclude that ants foraging collectively and interacting frequently, e.g. along foraging trails and at profitable food sources, remain in a social context and thereby maintain high aggressiveness against potential competitors. Our results suggest that the nestmate recognition system can be utilized at remote sites for an adaptive and flexible tuning of the response against competitors.

## Introduction

Securing resources often requires active defense against competing con- and heterospecifics. The most valuable resource of social insects is their nest, housing the reproductive individuals (queens), the developing and immobile brood, and food stores. Nests provide shelter from harsh environment and the opportunity for microclimatic control of the nest interior [1–3], but inevitably lead to a clustered occurrence of brood and food other species may prey on [4; 5]. Ants, bees and wasps vigorously defend their nests, and at the nest entrance workers that are engaged in patrolling and attacking potential intruders can be identified based on their behavior (e.g. guard bees [6]) or in several ant species even morphologically as soldiers with larger body size and distinct morphological traits [7–9].

In response to a threat, guarding and defending workers may use alarm pheromones to recruit nestmates [10–12]. In this scenario, perceiving the alarm pheromone influences the internal physiological state of the receiver (aggression context), making it more likely for the receiver to respond with aggression towards unfamiliar objects and intruders [11]. Such context-specific behavioral responses are common, either decreasing a response threshold or increasing a propensity for responding to a task-associated stimulus [13]. Besides the well described pheromonal communication, social interactions among workers and the nest environment have an impact on the individuals' internal state, changing it to a social context [11; 14].

While there are frequent interactions and signals among nestmates within the nest that may modulate a worker's context for an adequate behavioral response, comparable situations may occur at food sources [15]. Mass recruitment along pheromone trails and markings at highly profitable food sources result in aggregations and defense of these sites against conspecifics [16; 17] and heterospecifics [18; 19]. Since an individual ant itself might not be able to assess profitability of a resource, the aggressive defense of these remote 'jackpot sites' is surprising [20].

We hypothesize that frequent worker-worker interactions, e.g. mutual antennation and trophalaxis change the propensity of workers to respond aggressively against foreign workers. The nestmate recognition system that primarily functions for achieving colony coherence may play an important role in establishing a social context within the individual [21–25].

Ants can discriminate between nestmates and conspecific non-nestmates, using the odors found on the body surface [26; 27]. These odors consist of many species-specific low-volatile cuticular hydrocarbons (i.e. CHCs), which further become colony specific by environmental influences, e.g. diet and nest material [28–30]. An almost uniform, colony specific odor is achieved by frequent exchange of CHCs between nestmates, mostly through trophallaxis and allogrooming [31; 32].

Recognition of nestmates and discrimination from non-nestmates is achieved by phenotype matching of the CHC-profiles [33]. The concept behind is that ants compare the perceived CHC profile (one colony odor: label) with a neuronal representation (template) of their own colony odor, a process often termed label-template matching. Any mismatch between a label and template may be recognized as a non-nestmate [21].

It has to be noted that we are far from understanding the nature of the neuronal template and how label-template matching is achieved in the brain [34; 35]. Recent studies indicate that incomplete CHC profiles are sufficient for an acceptance and classification of nestmates (inclusion theory) [36; 37], and that perceptual differences among individuals may contribute to the collective response of defense [38; 39]. Colony coherence and the almost complete absence of aggression against colony members suggest a binary classification of nestmates vs non-nestmates. However, even within a colony, groups of nestmates can be recognized based on task-related changes of their CHC-profile by other ants, modifying their behavior and eventually leading to specific task allocation [40; 41]. Thus, nestmate recognition cues may influence the internal state of an individual (social context) and may also provide specific information about a recently performed tasks of an encountered worker.

In this study, we addressed the question whether the ant's social context affects aggressiveness, and we tested *Formica rufa* (Linneaus, 1761) workers for aggressive responses against non-nestmates in one-on-one encounters. In our reductionist approach, we excluded any territorial markings or the presence of food. The experimental groups of workers differed only in the amount of social interactions prior to the encounter with nestmates or non-nestmates in a test arena.

## Materials and methods

### Animals

For all experiments we used the territorially aggressive ant species *Formica rufa*. This species belongs to the IUCN Red List of Threatened Species (Category: near threatened. Social Insects Specialist Group. 1996. *Formica rufa*. e.T8645A12924924. http://dx.doi.org/10.2305/IUCN.UK.1996.RLTS.T8645A12924924.en. Downloaded on 11 July 2017). Permit for collection was issued by the “Untere Naturschutzbehörde, Regierungspräsidium Freiburg, BW, Germany; Befreiung nach § 45/7 BNatSchG”. Two colonies were collected in May 2012 near Markelfingen, BW, Germany (lat. 47°43’53.32”N, long. 9°1’51.42”E and lat. 47°43’53.17”N, long. 9°1’50.35”E, respectively). Both colonies were originally polygynous, and after establishing the colonies in the laboratory in artificial plaster nests with about 5000 workers each, colony 1 had two queens and colony 2 had one queen. The colonies were kept at a constant temperature of 25°C and 50%-60% humidity (12:12 h photoperiod), fed with honey water and frozen cockroaches twice a week and were provided with water ad libitum.

We decided on studying this Red-List species in order to obtain information that may aid protection measures, and also because we think that the underlying mechanism for resource defense is widespread across the majority of ant species.

### Behavioral assay

**Fig 1:**
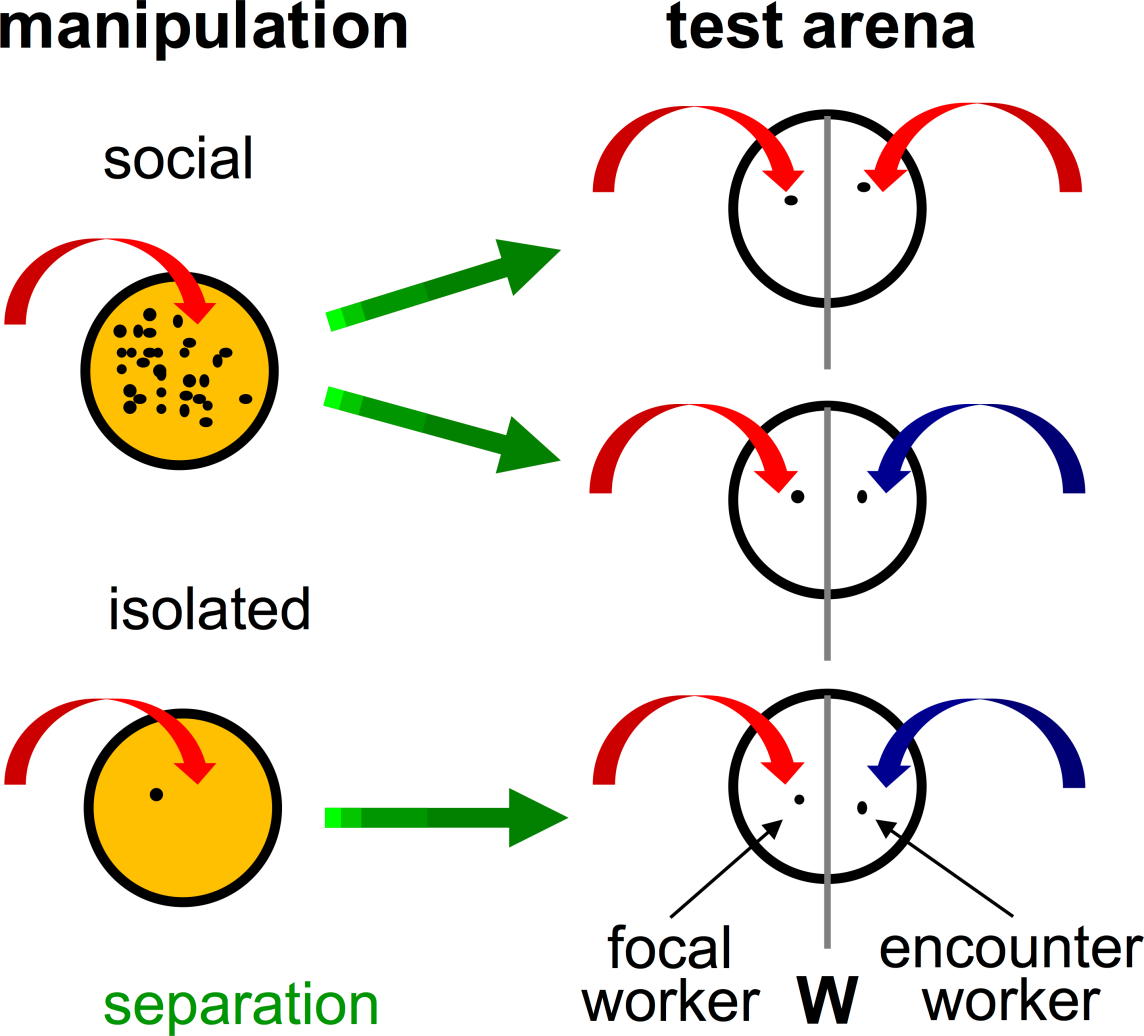
Experimental procedure. Workers were collected from the colony and transferred, first to one of the manipulation-arenas (red arrows) for either *social* or *isolated* manipulation. Later, after a *separation* time (green arrows), these focal-workers were transferred to one half of the test-arena (red arrows). Before the test started, a retractable separation wall (W) divided the test-arena into two halves. Encounter-workers were transferred to the other half of the test-arena, and either workers from the same colony (*NM*; red arrows) or a different colony (*NNM*; blue arrows) were used. Video recording and test started by sliding out the separation wall.

Workers were collected from a colony by branching them off from an exposed area located close to the nest. They climbed voluntarily onto a provided toothpick on which they were transferred into a plastic tube for cooling on ice until immobilized. This procedure prevented workers from releasing formic acid during the following procedure in which workers were placed either alone or together in groups of about 30 nestmates in a manipulation-arena with a Fluon coated wall and filter paper on the ground (diameter: 60 mm, height: 40 mm; Fig 1). Our manipulations were i) workers kept in isolation (*isolated*) and ii) workers kept in a social environment (*social*). After a separation time of 20 up to 286 min, workers from these manipulation-arenas were transferred, using a toothpick as before, to one side of a test-arena. The test-arena (diameter: 60 mm, height: 40 mm) was split in two halves by a separation wall, and was lined with fresh filter paper for each trial, thus, we excluded deposits of markings such as the trail pheromone. Over time, we transferred single workers successively from a social group, thereby reducing the number of workers in the group, and we stopped worker transfer when 20 workers were left in the manipulation-arena. This restriction ensured that all workers of social groups had frequent interactions before they were tested. Due to the small size of the manipulation-arena, workers in social groups had a high number of interactions. We decided on such a restricted space for the workers to promote interactions that maintain the social context for the workers, and this experimental design precluded us from quantifying individual interactions even within short time periods.

For testing, a single worker was directly transferred from one of the colonies to the other half of the test arena, and both workers were allowed to briefly acclimate to the new arena before the separation wall was removed (sliding sideways). The workers that came from the manipulation-arena were observed in detail (for a period of three minutes), and these workers are termed focal-workers in the following, while the other workers in each test are termed encounter-workers. All focal-workers came from colony 1 and encounter-workers either came from colony 2 (non-nestmates) or colony 1 (nestmates). The pairs of focal- and encounter-workers were size matched by visual inspection in order to reduce size-related bias in behavioral responses [42].

All individual data are independent from each other because workers were sacrificed after being tested. A total of 240 trials, consisting of pairs of focal- and encounter-workers were analyzed. The pairings for the tests (groupings) were: social focal-workers versus nestmates (*social-FW* vs *NM)*, social focal-workers versus non-nestmates (*social-FW* vs *NNM)* and *isolated-FW* vs *NNM* (n = 80 for each group), and tests were done in parallel on 19 days between 5^th^ June to 11^th^ July in the room were the colonies were kept.

Interactions between the two workers are defined by physical contact, such as antennation or targeted behavior of the focal-workers towards the encounter-worker (Table 1). In the vast majority of encounters, the focal-workers made antennal contact (in 2621 of a total of 2815 encounters in 240 trials), and encounters are terminated when the two workers separated spatially by at least one body length. Encounters that occurred after a worker (unsuccessfully) tried to escape by climbing the wall of the test-arena were excluded from the analysis.

**Table 1:** Behaviors of focal-workers while interacting with the encounter-worker during a trial.

behavior	description
antennation	antennae of focal-worker touch the encounter-worker at any body part
body rising	focal-worker raises its body while moving its front legs in the air
opening mandibles	mandibles of focal-worker are wide open and distance between opponents is less than one body length
**biting**	focal-worker grasps any body part of the opponent with its mandibles
**flexing gaster**	focal-worker bends its gaster forward, possibly spraying formic acid
**fighting**	both workers are entangled with each other, such that no further specific behaviors can be discriminated

The two workers were video recorded, using a high-speed camera (Casio, Exilim F1) at a sampling rate of 300 frames/sec for three minutes, starting with the removal of the separation wall. The videos were analyzed, using free video-editor software (Avidemux 2.5, Mean, fixounet.free.fr) and the behaviors of focal-workers were scored separately for all encounters throughout the trial (whole recording time of three minutes).

**Fig 2:**
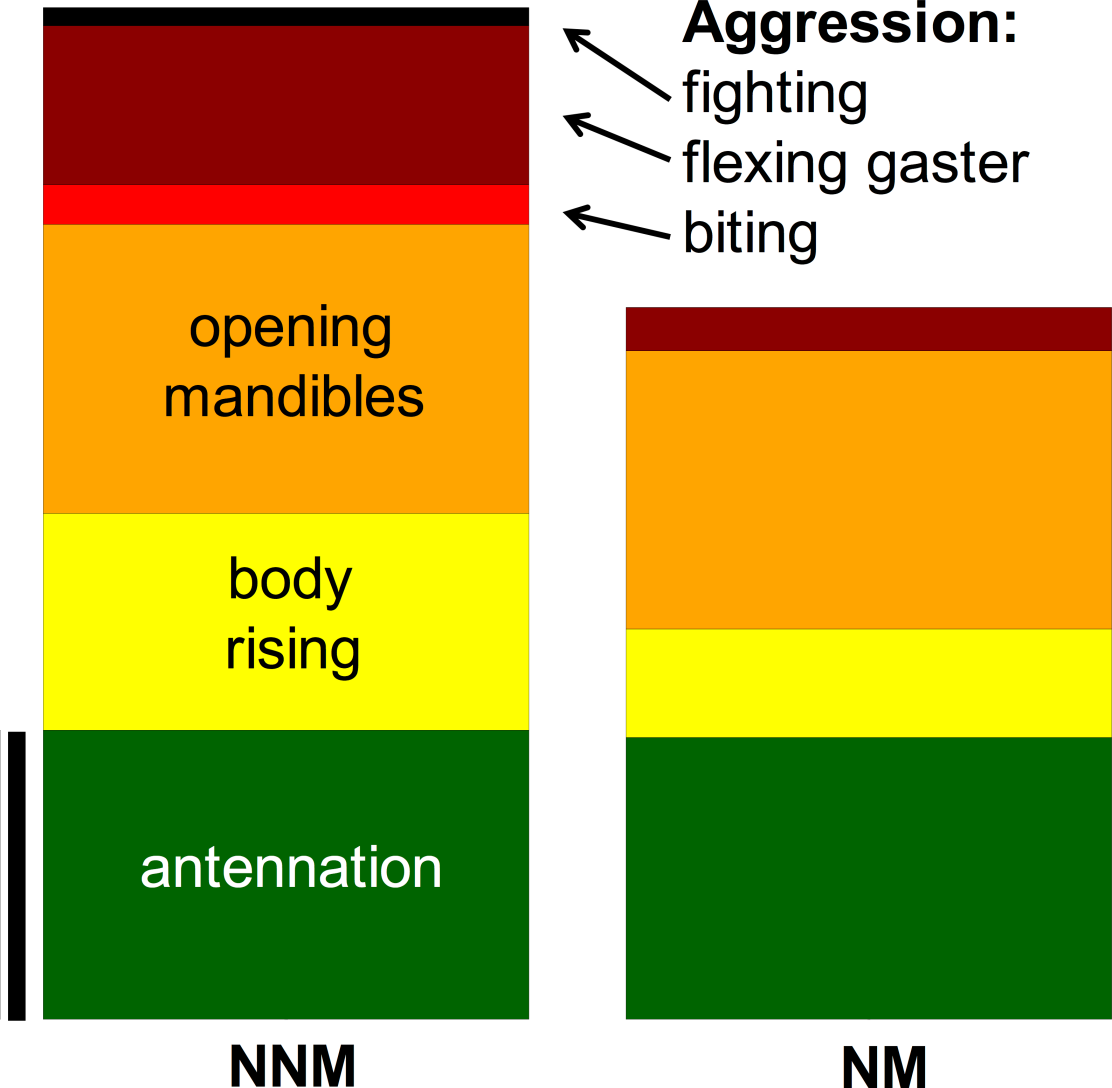
Behavioral scores of focal-workers for later binomial classification as being aggressive or not. Social focal-workers were examined with respect to behaviors that mainly or exclusively occurred in encounters with non-nestmates. If one of the following behaviors: 'biting', 'flexing gaster' or 'fighting' occurred at least once during any encounter, the focal-worker was classified as being aggressive. Otherwise, the focal-worker was classified as non-aggressive. Scale bar: 100% prevalence of a behavior in at least one of the encounters during a trial.

Our aim was to classify the behavior of focal-workers as being aggressive or non-aggressive, rather than describing aggressive behavior itself in detail. To this aim, we only scored unambiguous behaviors and abstained from an arbitrary renaming the video files for an observer-blind analysis. We built our classification scheme on focal-workers as being aggressive when they showed at least one of the clearly aggressive behaviors that are potentially harmful: 'biting', 'flexing gaster', 'fighting' in any of their encounters during a trial (Fig 2). The rational of this experimental procedure and scoring of behavior is to allow focal workers to experience the encounter-workers long enough to decide on aggression or not. Quantifying the odds of being aggressive based on our classification was 0.19 for *social-FW* vs *NM*, 1.58 for *social-FW* vs *NNM* and 0.38 for *isolated-FW* vs *NNM*. The low odds for *social-FW* vs *NM* confirms that our classification depicts specific aggression against *NNM*.

### Data analysis

First, we quantified potential differences between the groups in how many encounters (number of interactions) occurred. For comparisons, we used a quasi-Poisson generalized linear model (GLM), with groupings (*social-FW* vs *NM*, *social-FW* vs *NNM*, *isolated-FW* vs *NNM*) and the separation time as explanatory variable, and we also included an interaction of both variables (model I).

In order to draw inferences about differences between groups, we used a Bayesian framework by calculating 10 000 values that are random draws from the posterior distribution of the model I estimates. We compared the number of interactions at the shortest and longest separation time (20 min and 245 min) by the proportion of simulated values from the posterior distribution that are larger in one group compared to another group (measure of certainty). We specifically analyzed whether the isolation of focal-workers results in a disproportionate decrease in number of interactions which, in case, might impact our measure of aggression, for example: if the number of interactions decreases over time, this may lead to a lower number of focal-workers being classified as being aggressive.

Second, we tested whether the number of interactions impacts our measure of aggression. We used a binomial GLM (model II), with a logit link function to quantify differences in aggression of focal-workers in response to *NM* and *NNM*. To the binary response variable of aggression, we included number of interactions as well as grouping and their interactions as explanatory variables. As in the Bayesian framework above, we calculated 10 000 values that are random draws from the posterior distribution of the model II estimates for the description of the credible intervals.

Third, in the following model we excluded the number of interactions since they had no impact on our measure of aggressiveness (model II). We used a binomial GLM (model IIIA), with a logit link function to quantify differences in aggression of focal-workers in response to *NM* and *NNM*. To the binary response variable of aggression, we included separation time (log-transformed), grouping and their interactions as explanatory variables. Based on the model estimates, we calculated the probability of being aggressive at short (20 min) and long (245 min) separation times, and we calculated the change in odds over time for being aggressive.

As in the Bayesian framework above, we calculated 10 000 values that are random draws from the posterior distribution of the model IIIA estimates. We compared the probability of aggression at the shortest and longest separation time (20 min and 245 min) by the proportion of simulated values from the posterior distribution that are larger in one group compared to another group (measure of certainty).

Finally, using the same approach, we compared the change over time in being aggressive between *isolated-FW* and *social-FW* vs *NNM* and between *social-FW* vs *NM* and *NNM*. In addition, we estimate the effect of separation time on the probability of workers being aggressive by using a linear regression (model IIIB) on the simulated data from model IIIA.

In the Bayesian statistics, we always used the 2.5% and the 97.5% quantiles as the lower and the upper limits of the 95% credible interval. All statistical analyses were done using R (3.3.3) [43] in RStudio, including the package *arm* with the *sim* function to draw random samples from the posterior distribution of model parameters [44].

## Results

**Fig 3:**
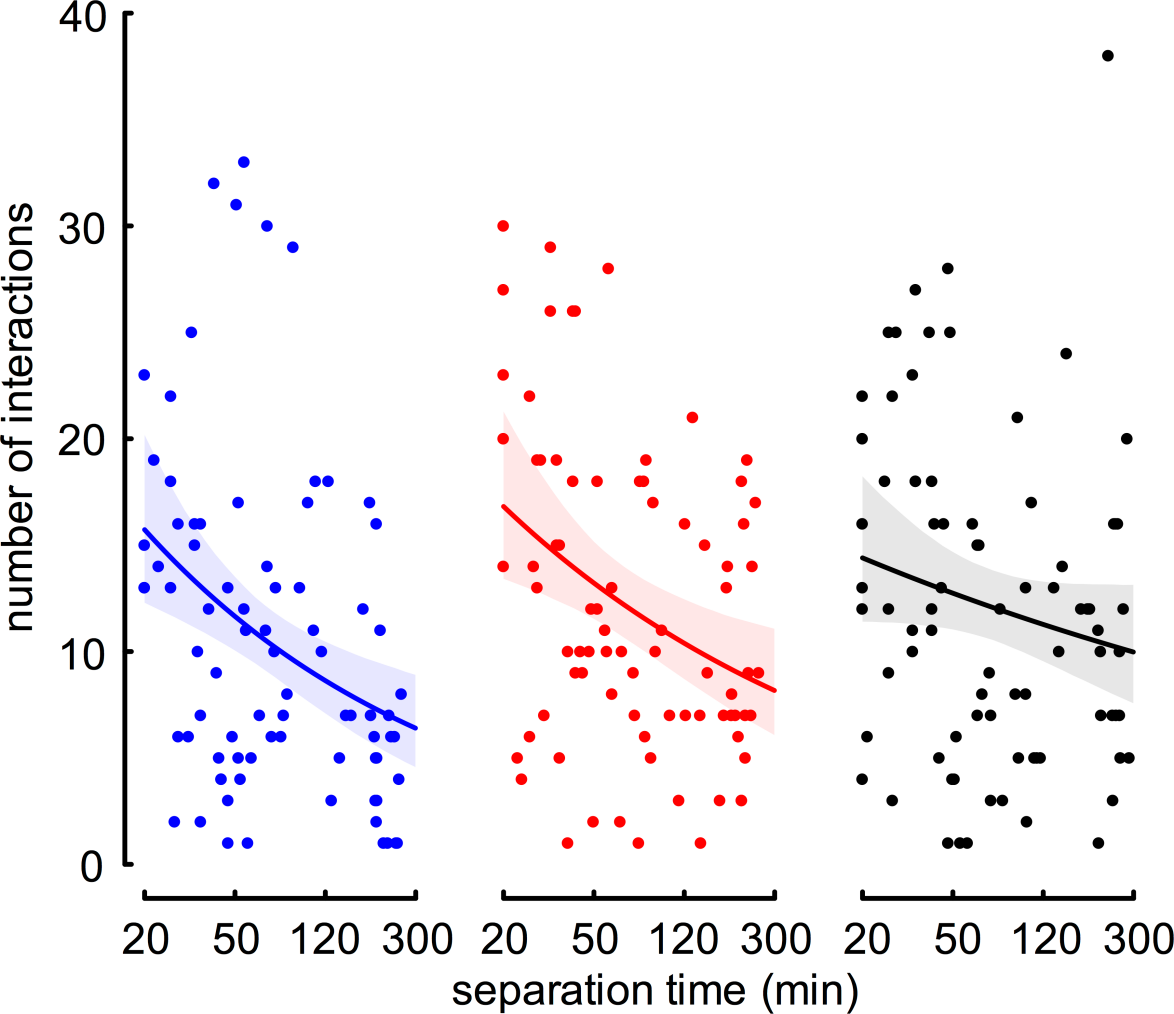
Effect of separation time (min) on number of interactions. In two of the groups (*social-FW* vs *NM* in blue: -28% and *social-FW* vs NNM in red: -24%), the number of interactions decreases significantly with increasing separation time. For the group of *isolated-FW* vs *NNM* (black: -13%), this decrease is lower and statistically not significant different from no change. Dots represent independent measures of number of interactions. Solid lines represent fitted values from model I and shaded areas represent the 95% credible intervals of Bayesian statistics.

Focal-workers of all three groups interacted frequently with their encounter-workers (Fig 3). Irrespective of separation time, the median number of interactions was very similar across the groups (*social-FW* vs *NM*: 8.5, quartile range (QR): 5–15; *social-FW* vs *NNM*: 10, QR: 7–18; *isolated-FW* vs *NNM*: 12, QR: 6–16).

We calculated the correlation between separation time and number of interactions, using the GLM model I (S1 Table). At the beginning of our observation period, at a separation time of 20 min, the calculated numbers of interactions are 16 for *social-FW* vs *NM*, 17 for *social-FW* vs *NNM* and 14 for *isolated-FW* vs *NNM* (Fig 3). At longer separation time, the number of interactions decreased significantly in focal-workers from social groups, irrespectively whether they encounter a *NM* or a *NNM* (decrease by 28% and 24% for one log-unit increase in time, respectively). The focal-workers that previously were kept in isolation also had fewer interactions at longer separation time, however, this decrease by 13% does not differ significantly from no change (S1 Table).

Bayesian statistics revealed low certainty of differences between number of interactions at short separation times (20 min; 65% between the two groups of *social-FW* and 82% for *isolated-FW* vs *NNM*, having fewer interactions compared to *social-FW* vs *NNM*). We find no indication that after long separation time (245 min) *isolated-FW* have fewer interactions compared to the two social groups. Rather the opposite seems to be the case: with certainties of 98% and 83% (*social-FW* vs *NM* and vs *NNM*, respectively), *isolated-FW* had more interactions at longest separation time.

The decrease in number of interactions (slope) is very similar between the two social groups, *social-FW* vs *NM* and *social-FW* vs *NNM* (certainty of difference is only 69%), and it is very unlikely that the decrease in number of interactions is higher in *isolated-FW* vs *NNM* compared to the two groups of *social-FW* (14% and 5% for *social-FW* vs *NNM* and vs *NM*, respectively). We therefore are confident that our measure of aggression is not biased, thus not favoring our hypothesis by the number of interactions at different separation time.

Since the number of interactions relates to separation time in some of the groups tested, we further analyzed this parameter as explanatory variable for aggression. In all three groups, the number of interactions has no significant predictive value for the probability of an aggressive response in focal-workers. A larger number of interaction is not related to a higher probability for being classified as aggressive, since we found no significant change in odds. The calculated change in odds from the GLM (model II) are: 2% for *social-FW*, irrespective of the type of encounter (*NM* or *NNM*) and 4% for *isolated-FW* against *NNM* for each additional interaction (S2 Table and S1 Fig), and we found large and overlapping credible intervals.

Based on the results that: i) number of interactions does not relate to the probability of *isolated-FW* being classified as aggressive (model I) and ii) *isolated-FW* have the lowest decrease in number of interactions with longer separation time (model II), we decided to exclude the number of interactions as explanatory variable in the following model.

**Fig 4:**
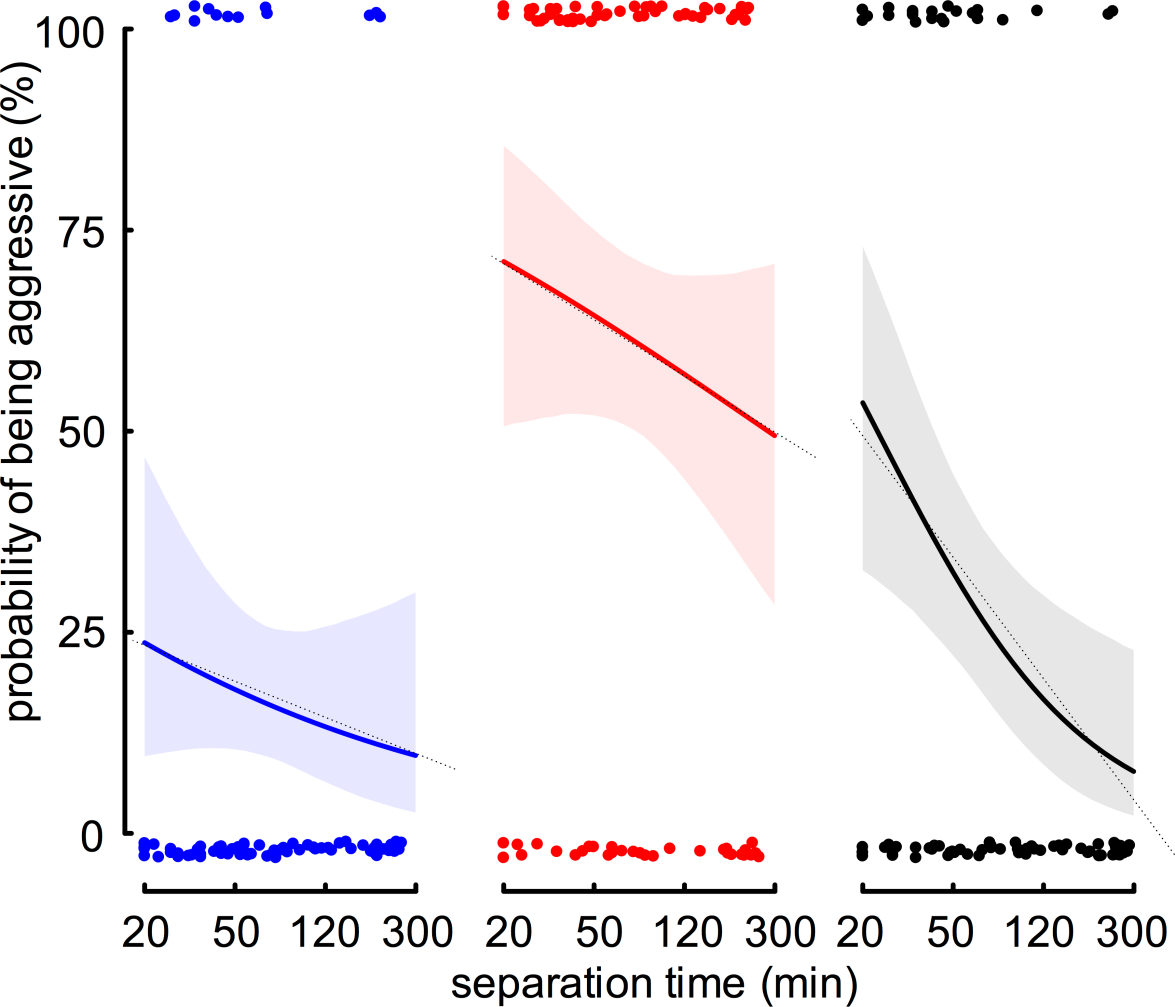
Effect of separation time (min) on probability of being aggressive. *Social-FW* differ with high certainty (>99.9%) in their probability of being aggressive at short separation time (20 min), depending on the type of encounter ant (vs *NM* in blue: 24% and vs *NNM* in red: 71%). The change in odds decreases with increasing separation time, and this decrease is only significant for *isolated-FW* (vs *NNM* in black: -62%). Dots represent binary, independent data of focal-workers that were classified either as being aggressive (upper) or non-aggressive (lower). Solid lines represent fitted values from the binomial GLM (model IIIA) and shaded areas represent the 95% credible interval (based on Bayesian statistics). Dotted lines are linear regressions on log-transformed separation times (model lllB).

We calculated the correlation between separation time and probability of being aggressive, using the GLM model IIIA. Over separation time, the probability of being aggressive changes (Fig 4). The calculated change in odds for being aggressive from model IIIA are: -33% for *social-FW* vs *NM*; -29% for *social-FW* vs *NNM* and -62% for *isolated-FW* vs *NNM* for one log-unit increase in time. Only the change in odds of *isolated-FW* is significantly different from zero (S3 Table).

The estimates for the intercepts (probability of aggression at separation time equal zero) are imprecise because of missing data during the period of 0–20 min of separation time. Only the (predicted) aggression at a separation time of 0 min of *isolated-FW* vs *NNM* are significantly different from no aggression. At a separation time of 20 min, we calculated the lowest probability of being aggressive for *social-FW* vs *NM* and the highest probability of being aggressive for *social-FW* vs *NNM* (24% and 71%, respectively, and 54% for *isolated-FW* vs *NNM*). At long separation times (245 minutes), the probability of being aggressive drops down in *isolated-FW* vs *NNM* and is comparably low in *social-FW* vs *NM* (9% and 10%, respectively) but remains high in *social-FW vs NNM* (51%).

Bayesian statistics revealed high certainty of differences between *social-FW* vs *NM* and *social-FW* vs *NNM* at separation time 20 min (99.9%), thus, the type of encounter worker determines the focal-worker's response. This is also supported by the certainty of difference between *social-FW* vs *NM* and isolated-*FW* vs *NNM* (97%). The certainty of difference between *social-FW vs NNM* and *isolated-FW vs NNM* is less than 90%, indicating that these two groups do not differ considerably when the manipulation (social/isolated) lasted only 20 min.

After a long separation time (245 min), the difference between the two groups of *social-FW* vs *NM* and *NNM* is maintained (certainty of 99.8%). Now also the *isolated-FW* differ with high certainty (99.9%) from the *social-FW* vs *NNM* and this marked difference is the result of our manipulation. Following a long separation time, *isolated-FW* have a similar probability of being aggressive against *NNM* as *social-FW* against *NM* (certainty of difference only 55%).

Further support for our hypothesis that isolation reduces the probability of being aggressive against *NNM* is provided by the comparison of the change in aggression over time. The decrease in the probability of being aggression is, with a certainty of more than 90%, stronger in *isolated-FW* compared to *social-FW* against *NNM*.

In order to estimate the change in probability of being aggressive in the separation time-range we investigated, we calculated a linear regression (LM; model IIIB), using simulated values from Bayesian statistics that are based on data from the previous GLM (model IIIA). The decrease in probability of an aggressive response is at least two times higher in *isolated-FW* compared to *social-FW* (*social-FW* vs *NM*: -5%, *social-FW* vs *NNM*: -8%, *isolated-FW* vs *NNM*: -17% change for one log-unit increase in time).

## Discussion

The behavior of individual workers within an insect colony depends strongly on their social environment. In this study, we investigated how the social environment modulates aggression, and we show that a social environment maintains or generates a worker’s context that facilitates an aggressive response against potential competitors. Without a social environment, e.g. isolated from nestmates, workers have a reduced propensity for an aggressive response against workers from a different colony. We propose that nestmate recognition, a system primarily functioning for colony coherence, modulates individual aggressiveness. Aggregations of workers, for example at highly profitable food sources after mass recruitment along with frequent social interactions can promote a switch from foraging to defense behavior.

The defense of highly profitable food sources is a well-established phenomenon in ants [16; 45], and in our reductionistic approach we excluded any other cues that may occur at a feeding site, and focused on the effect of the presence or absence of a social environment for different time spans, and how this effects the probability of being aggressive to a non-nestmate. We based our classification on behaviors that are obviously threatening, and this conservative classification of aggression excludes the ‘spreading the mandibles’ that is used in many other studies as indicative for recognition of a non-nestmate [46]. Since our focal observations were long, we observed the 'spreading the mandibles' in almost all trials at least once, and this behavior was not necessarily triggered by the encounter-workers.

Aggressive interactions during an encounter depend on both workers participating. An aggressive behavior by one of the workers may lead to either an escalation, where both workers are engaged in aggression, or retraction, where one worker may disengage from the interaction. Thus, the encounter-workers in our tests probably contribute to the focal-worker’s response by initiating aggressive interactions. This may lead to an overestimation of focal-workers' aggressiveness against *NNM* encounter-workers and, if amicable behavior also is influential, to an even lower aggressiveness towards *NM* encounter-workers (false positive response). The main result of our study is based on the separation time of the focal-workers and the corresponding *NNM* encounter-workers are expected to contribute in the same way to the focal-worker's behavior, irrespective of their separation time since encounter-workers were all treated equally.

The CHC-profiles of workers possibly change while foraging, similar to what has been described for patrolling harvester ants [22; 25; 47]. However, it is clearly not rendered to a non-nestmate CHC-profile, since homecoming foragers are readily accepted by their nestmates even after longer absences. In our (control) experiments, even a separation from the colony for 4 hours did not impair nestmate recognition to a greater extent. We cannot rule out that during separation slight changes in the CHC-profile might occur, but it is highly unlikely that these changes could contribute significantly to reduced aggression in encounter-workers, and in turn in focal-workers. Thus, we are confident that the differences in behavior we observed in focal-workers are due to differences in their own social context rather than due to changes in their CHC-profile and corresponding behavioral changes in encounter-workers.

Other forms of communication, e.g. pheromonal signals and chemical markings have received much more attention in the field of task allocation in social insects than the social environment and interactions. Chemical markings are used in many ant species to spatially extend the nest environment with a home range or a territory [48–51]. For example, *Tetramorium* species mark their foraging area (i.e. home range), resulting in an increased probability of aggressive behavior against heterospecific, but not against conspecific non-nestmate workers [51]. In contrast to home range markings, territorial markings can promote aggression against non-nestmates [11]. Another fascinating example of competition among conspecific colonies is the ritualized combat in *Myrmecocystus mimicus*. During display tournaments that may last for several days, in which almost no physical fights occur, workers of two different colonies gauge each other and assess group size by 'head-counting', 'caste polling', and 'presence of opponents uninvolved in display fights' have been proposed [5; 52]. The underlying mechanisms for such an assessment are unknown. Based on our results, the absolute and relative rate of interactions with nestmates and non-nestmates probably contributes considerably to the modulation of a worker’s propensity for an aggressive response. Interestingly, ‘body rising’ occurred more often in our experiments with non-nestmates and it is also the common behavioral display of workers engaged in the tournament. Further support for the idea that beside the absolute number of nestmates also the relative number of nestmates vs. non-nestmates and interactions among them has an influence on the outcome in competitions comes from studies on Azteca ants when territories are formed [53].

Accumulations of large numbers of workers do not only occur within and close to the nest or in display tournaments, but also at valuable food sources as a consequence of mass recruitment along pheromone trails [54; 55]. In a previous study, in which workers were collected from foraging trails and tested against heterospecific workers, the authors already speculated that recent experience with nestmates may modulate the workers' aggressiveness [20]. Frequent social interactions (e.g. antennation, trophallaxis, or allogrooming) occur wherever many workers aggregate. During encounters with nestmates, workers are exposed to and perceive the CHC-profile of nestmates. For *Formica xerophila*, the exposure to nestmate CHC-profiles (presented on glass beads) maintains a high aggressiveness against heterospecific workers [18; 19; 56]. Our study extends this finding by demonstrating that the workers’ context is modulated such that task allocation for defense in general (not only against heterospecifics) is facilitated.

In solitary foraging species, e.g. in the desert ant *Cataglyphis fortis*, the individuals’ homing vector modulates the worker’s context, with long homing vectors relating to low and short homing vectors relating to high propensity of aggression [57]. Desert ants have to rely on their (egocentric) homing vector as an indication for vicinity to their home, as odor cues can be too volatile in the extreme desert habitat. Indeed, social interactions are not necessary to maintain aggressiveness against non-nestmates in the desert ant *C*. *niger*, whereas learning the CHC-profiles of familiar, neighboring conspecific colonies impacts aggressiveness [58]. Such colony level 'dear enemy' effects as well as 'nasty neighbor' effects have also been described in other ant species. Genetic, as well as spatial and chemical differences can lead to various, causal relationships among colonies and their aggressiveness [59–62].

**Fig 5:**
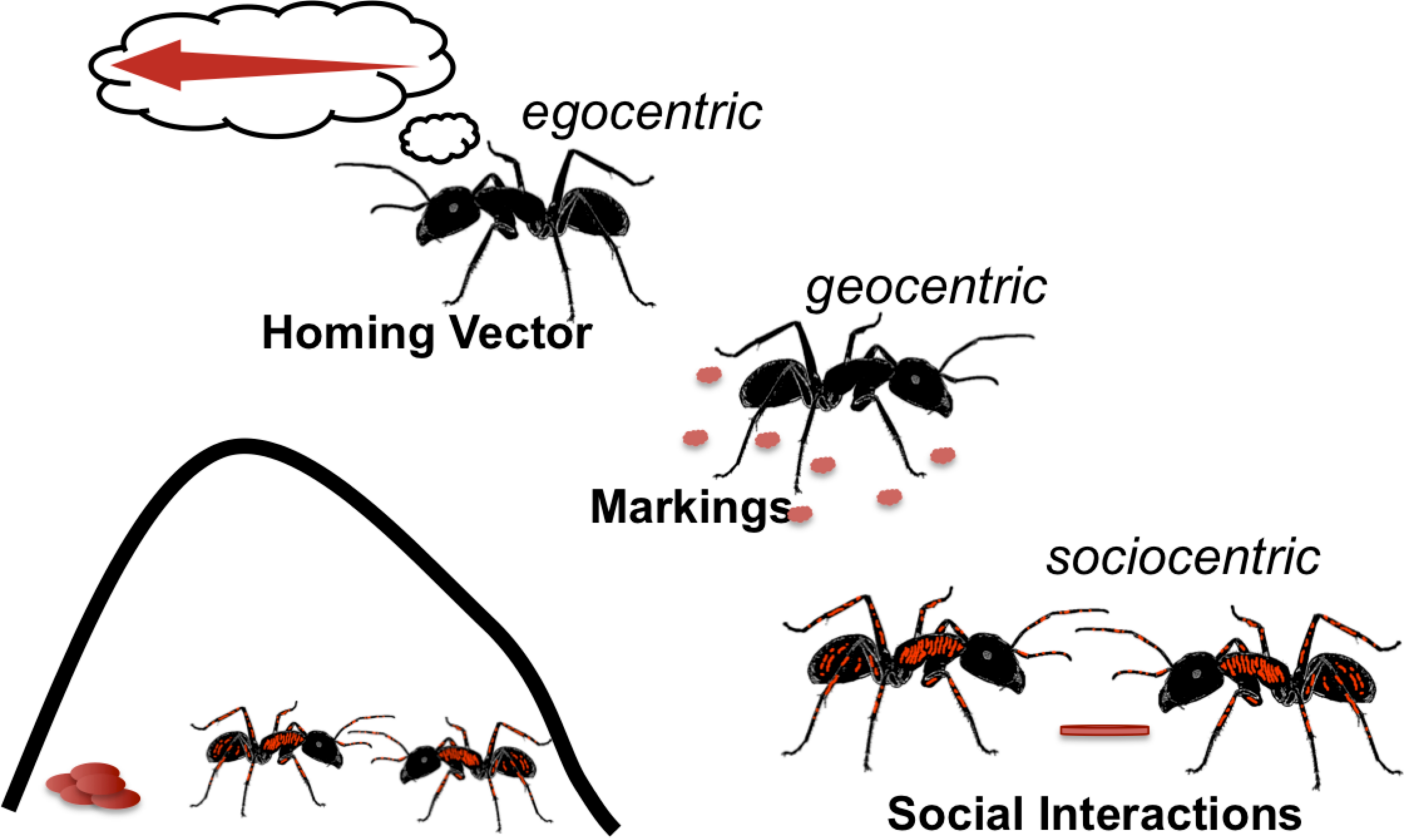
Worker's context promoting aggressive responses. The nest environment provides various cues that might modulate a worker’s context and eventually promotes aggression against competing con- and heterospecifics. A sociocentric modulation of defense of highly profitable food sources is plausible, when many foraging workers are gathering at the source. Some ant species use chemical markings, indicating the home range and areas with potentially valuable resources, which are defended mainly against heterospecifics [50]. Such a geocentric modulation of context is more stable over time and less flexible than a sociocentric modulation. Solitary foragers like the desert ant *Cataglyphis* can access the homing vector of their path integration, and the length of the homing vector is negatively correlated with the social context of the forager for an aggressive response [57; 63]. Such egocentric modulation of context seems most important for species without mass recruitment but might also act together with geocentric and sociocentric modulation in other species.

Although conclusions from reductionistic experimental approaches might suggest something else, egocentric, geocentric and sociocentric modulation of social context probably occurs in parallel at all times with the consequence of a fine-tuned task allocation within the colony. Except for desert ants that rarely deploy trail pheromones or other chemical markings and rarely encounter nestmates while foraging, other ants most likely use all these available cues that reliably indicate a social environment. An egocentric modulation of context, based solely on the homing vector will not lead to a collective defense at remote food sources, while a geocentric modulation with chemical markings can induce this (Fig 5). Along a pheromone trail, the modulation is directly related to recruitment and aggregation of workers, and it may even support protection of resources while transported. Marking of a territory rather reflects the colony's experience in the past, which might have predictive value for upcoming resources in the same area. A sociocentric modulation, based on interacting workers is a direct measure of the social environment and its momentariness makes it highly flexible. Species might be biased towards one available cue over another, depending on adaptations to different habitats, colony organization and life history.

In general, context is a neurophysiological state and any modulation of the context is an internal process leading to a different state. The classical candidates for neuromodulation are biogenic amines, like dopamine, octopamine and serotonin [64–66].

In order to fill our gap in understanding the link between perception (e.g. non-nestmate) and action (e.g. aggressive response), we need to investigate the underlying neural mechanisms that lead to individual decision making. The ease of manipulating an individual's social context and assessing aggressive behavior makes the nestmate recognition system of ants a highly promising system for experimentally addressing both, proximate mechanisms and the consequences of individual behavior on the social organization in insect colonies.

## Supporting information

S1 Fig Effect of number of interactions on probability of being aggressive. With larger numbers of interactions, there is no significant increase in the probability of being aggressive (flaring credible intervals). When having few interactions within a trial, social-FW were more likely to act aggressively against NNM compared to encounters with NM (significant difference in intercepts). This indicates that only very few interactions are necessary to discriminate NNM from NM. Dots represent binary, independent data of focal-workers that were classified either as being aggressive (upper) or non-aggressive (lower). Solid lines represent fitted values from model II and shaded areas represent the 95% credible intervals of Bayesian statistics.
(TIFF)

S1 Table Results of GLM model I: Correlation between separation time and number of interactions. glm(formula = NoInt ~ grouping + time.log + time.log:grouping, family = quasipoisson, data = data.glm). Estimates can be back-transformed using exp().
(DOCX)

S2 Table Results of GLM model II: Correlation between number of interactions and probability of being aggressive. glm(formula = aggression ~ NoInt * grouping, family = binomial(link = "logit"), data = data.glm). Estimates can be back-transformed using plogis().
(DOCX)

S3 Table Results of GLM model IIIA: Correlation between separation time and probability of being aggressive. glm(formula = aggression ~ grouping + time.log + time.log:grouping, family = binomial(link = "logit"), data = data.glm). Estimates can be back-transformed using plogis().
(DOCX)

S1 Dataset Spreadsheet with raw data. These data were used to generate S2 Dataset.
(CSV)

S2 Dataset Spreadsheet with pre-processed data. These data correspond to data.glm.
(CSV)
