# Supplementary figures and images for: Social interactions promote adaptive resource defense in ants
Source: PLoS One. 2017 Sep 14;12(9):e0183872. doi: 10.1371/journal.pone.0183872 (PMC5598949; doi:10.1371/journal.pone.0183872)

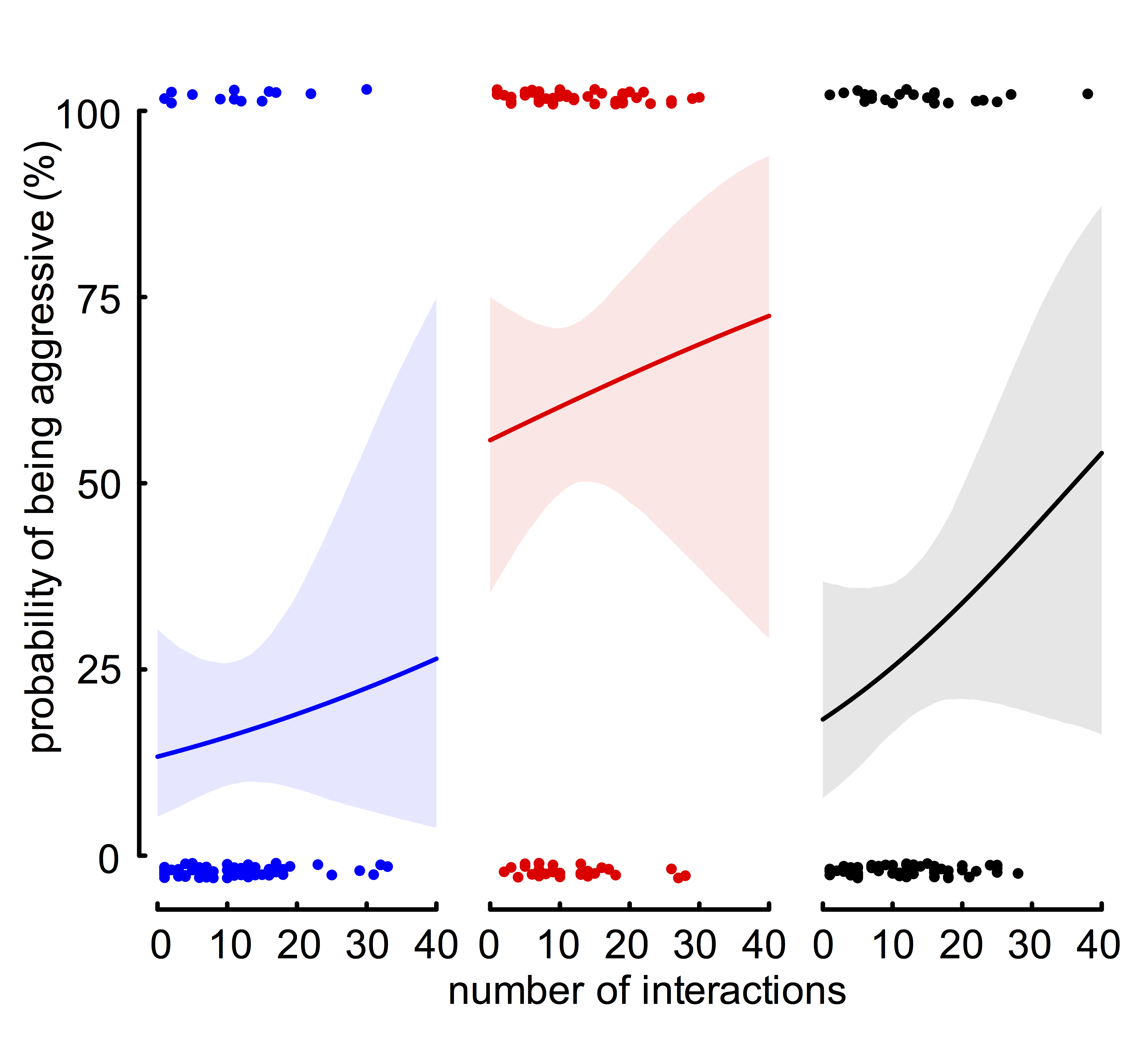

Supplement: S1 Fig — With larger numbers of interactions, there is no significant increase in the probability of being aggressive (flaring credible intervals). When having few interactions within a trial, social-FW were more likely to act aggressively against NNM compared to encounters with NM (significant difference in intercepts). This indicates that only very few interactions are necessary to discriminate NNM from NM. Dots represent binary, independent data of focal-workers that were classified either as being aggressive (upper) or non-aggressive (lower). Solid lines represent fitted values from model II and shaded areas represent the 95% credible intervals of Bayesian statistics. (TIFF) [file pone.0183872.s001.tiff]
