# Supplementary material for: Social interactions promote adaptive resource defense in ants
Source: PLoS One. 2017 Sep 14;12(9):e0183872. doi: 10.1371/journal.pone.0183872 (PMC5598949; doi:10.1371/journal.pone.0183872)
Supplement: S1 Table — glm(formula = NoInt ~ grouping + time.log + time.log:grouping, family = quasipoisson, data = data.glm). Estimates can be back-transformed using exp(). (DOCX) [file pone.0183872.s002.docx]

**Estimates of intercepts and slopes, separately for each of the three groups**

|  | **Estimate** | **Std. Error** | **t value** | **Pr(>\|t\|)** |
| --- | --- | --- | --- | --- |
| intercept social-FW vs NNM | 3.6241 | 0.3625 | 9.9971 | 0.0000 |
| slope social-FW vs NNM | -0.2673 | 0.0865 | -3.0896 | 0.0022 |
| intercept isolated-FW vs NNM | 3.0749 | 0.3487 | 8.8185 | 0.0000 |
| slope isolated-FW vs NNM | -0.1359 | 0.0814 | -1.6697 | 0.0963 |
| intercept social-FW vs NM | 3.7539 | 0.3948 | 9.5081 | 0.0000 |
| slope social-FW vs NM | -0.3329 | 0.0953 | -3.4945 | 0.0006 |

**Contrast estimates of the reference group (social vs NM) compared to the other two groups**

|  | **Estimate** | **Std. Error** | **t value** | **Pr(>\|t\|)** |
| --- | --- | --- | --- | --- |
| Intercept social-FW vs NM | 3.7539 | 0.3948 | 9.5081 | 0.0000 |
| to intercept social-FW vs NNM | -0.1298 | 0.5360 | -0.2422 | 0.8089 |
| to intercept isolated-FW vs NNM | -0.6790 | 0.5267 | -1.2891 | 0.1986 |
| slope social-FW vs NM | -0.3329 | 0.0953 | -3.4945 | 0.0006 |
| to slope social-FW vs NNM | 0.0656 | 0.1287 | 0.5099 | 0.6106 |
| to slope isolated-FW vs NNM | 0.1970 | 0.1253 | 1.5726 | 0.1172 |

**Deviance Residuals:**

| Min | 1Q | Median | 3Q | Max |
| --- | --- | --- | --- | --- |
| -4.59 | -1.68 | -0.37 | 1.16 | 6.61 |

(Dispersion parameter for quasipoisson family taken to be 4.499142)

Null deviance: 1182.5 on 239 degrees of freedom

Residual deviance: 1058.2 on 234 degrees of freedom

AIC: NA

Number of Fisher Scoring iterations: 5
