# Supplementary material for: Social interactions promote adaptive resource defense in ants
Source: PLoS One. 2017 Sep 14;12(9):e0183872. doi: 10.1371/journal.pone.0183872 (PMC5598949; doi:10.1371/journal.pone.0183872)
Supplement: S2 Table — glm(formula = aggression ~ NoInt * grouping, family = binomial(link = "logit"), data = data.glm). Estimates can be back-transformed using plogis(). (DOCX) [file pone.0183872.s003.docx]

**Estimates of intercepts and slopes, separately for each of the three groups**

|  | **Estimate** | **Std. Error** | **z value** | **Pr(>\|z\|)** |
| --- | --- | --- | --- | --- |
| intercept social-FW vs NNM | 0.2352 | 0.4474 | 0.5257 | 0.5991 |
| slope social-FW vs NNM | 0.0184 | 0.0321 | 0.5744 | 0.5657 |
| intercept isolated-FW vs NNM | -1.4934 | 0.4980 | -2.9985 | 0.0027 |
| slope isolated-FW vs NNM | 0.0414 | 0.0327 | 1.2661 | 0.2055 |
| intercept social-FW vs NM | -1.8757 | 0.5247 | -3.5748 | 0.0004 |
| slope social-FW vs NM | 0.0213 | 0.0372 | 0.5733 | 0.5665 |

**Contrast estimates of the reference group (social vs NM) compared to the other two groups**

|  | **Estimate** | **Std. Error** | **z value** | **Pr(>\|z\|)** |
| --- | --- | --- | --- | --- |
| intercept social-FW vs NM | -1.8757 | 0.5247 | -3.5750 | 0.0004 |
| to intercept social-FW vs NNM | 2.1109 | 0.6896 | 3.0610 | 0.0022 |
| to intercept isolated-FW vs NNM | 0.3823 | 0.7234 | 0.5280 | 0.5972 |
| slope social-FW vs NM | 0.0213 | 0.0372 | 0.5730 | 0.5665 |
| to slope social-FW vs NNM | -0.0029 | 0.0491 | -0.0590 | 0.9531 |
| to slope isolated-FW vs NNM | 0.0201 | 0.0495 | 0.4060 | 0.6846 |

**Deviance Residuals:**

| Min | 1Q | Median | 3Q | Max |
| --- | --- | --- | --- | --- |
| -1.51 | -0.78 | -0.58 | 1 | 2 |

(Dispersion parameter for binomial family taken to be 1)

Null deviance: 310.77 on 239 degrees of freedom

Residual deviance: 269.67 on 234 degrees of freedom

AIC: 281.67

Number of Fisher Scoring iterations: 4
