# Supplementary material for: Social interactions promote adaptive resource defense in ants
Source: PLoS One. 2017 Sep 14;12(9):e0183872. doi: 10.1371/journal.pone.0183872 (PMC5598949; doi:10.1371/journal.pone.0183872)
Supplement: S3 Table — glm(formula = aggression ~ grouping + time.log + time.log:grouping, family = binomial(link = "logit"), data = data.glm). Estimates can be back-transformed using plogis(). (DOCX) [file pone.0183872.s004.docx]

**Estimates of intercepts and slopes, separately for each of the three groups**

|  | **Estimate** | **Std. Error** | **z value** | **Pr(>\|z\|)** |
| --- | --- | --- | --- | --- |
| intercept social-FW vs NNM | 1.9318 | 1.2784 | 1.5111 | 0.1308 |
| slope social-FW vs NNM | -0.3420 | 0.2901 | -1.1789 | 0.2384 |
| intercept isolated-FW vs NNM | 3.0602 | 1.4353 | 2.1321 | 0.0330 |
| slope isolated-FW vs NNM | -0.9719 | 0.3524 | -2.7576 | 0.0058 |
| intercept social-FW vs NM | 0.0133 | 1.6612 | 0.0080 | 0.9936 |
| slope social-FW vs NM | -0.3941 | 0.3971 | -0.9926 | 0.3209 |

**Contrast estimates of the reference group (social vs NM) compared to the other two groups**

|  | **Estimate** | **Std. Error** | **z value** | **Pr(>\|z\|)** |
| --- | --- | --- | --- | --- |
| Intercept social-FW vs NM | 0.0133 | 1.6612 | 0.0080 | 0.9936 |
| to intercept social-FW vs NNM | 1.9186 | 2.0962 | 0.9153 | 0.3600 |
| to intercept isolated-FW vs NNM | 3.0469 | 2.1954 | 1.3879 | 0.1652 |
| slope social-FW vs NM | -0.3941 | 0.3971 | -0.9926 | 0.3209 |
| to slope social-FW vs NNM | 0.0521 | 0.4917 | 0.1060 | 0.9156 |
| to slope isolated-FW vs NNM | -0.5778 | 0.5309 | -1.0882 | 0.2765 |

**Deviance Residuals:**

| Min | 1Q | Median | 3Q | Max |
| --- | --- | --- | --- | --- |
| -1.58 | -0.74 | -0.51 | 0.95 | 2.18 |

(Dispersion parameter for binomial family taken to be 1)

Null deviance: 310.77 on 239 degrees of freedom

Residual deviance: 260.51 on 234 degrees of freedom

AIC: 272.51

Number of Fisher Scoring iterations: 4
